# Past and Future Perspectives for Hepatitis B and C in Pakistan

**DOI:** 10.7759/cureus.17521

**Published:** 2021-08-28

**Authors:** Sarmad Zahoor, Aleena Khan, Sadia Asif, Sair Ahmad Tabraiz, Hossam Mustafa, Sheraz Ansar, Sumera Hanif, Hassan Ali Raza

**Affiliations:** 1 Medicine, Punjab Institute of Cardiology, Lahore, PAK; 2 Internal Medicine, Allama Iqbal Medical College, Jinnah Hospital, Lahore, PAK; 3 Rheumatology, Fatima Memorial Hospital, Lahore, PAK; 4 Internal Medicine, Mansoura University, Mansoura, EGY; 5 Internal Medicine, Gujranwala Medical College, District Headquarter Teaching Hospital, Gujranwala, PAK; 6 Internal Medicne, Allama Iqbal Medical College, Jinnah Hospital, Lahore, PAK

**Keywords:** least squares regression, future burden, blood donors, hcv, hbv

## Abstract

Background

Hepatitis B and C are viral infections of the liver transmitted by blood contamination. These infections are endemic in Pakistan and put a tremendous burden on its healthcare system. We conducted this study to assess the prevalence of hepatitis B virus (HBV) and hepatitis C virus (HCV) infections in Gujranwala, Pakistan, from 2010 to 2015 and determine the trend of future infections for a prediction of the disease burden by 2030 so policymakers can make informed decisions.

Methods

We conducted a retrospective cross-sectional study of 66,308 healthy blood donor samples at District Headquarters Teaching Hospital in Gujranwala from January 2010 to December 2015. Samples were screened for HBV and HCV using the kit method, and data were analyzed using IBM SPSS Statistics for Windows, version 20.0 (IBM Corp., Armonk, NY). We applied a least squares regression to our results to predict HBV and HCV incidence in 2030.

Results

A total of 715 samples (1.08%) were positive for HBV and 1,846 samples (2.78%) were positive for HCV. Our projections indicate that 3.25% of patients in Pakistan will be positive for HBV, and 6.36% will be positive for HBC by 2030.

Conclusion

We found an unexpectedly greater burden of HBV and HCV in the recent past than at current levels. The predicted percentages of future burden over the next decade were alarmingly high. These data necessitate implementing preventive and therapeutic measures by policymakers to reduce the disease burden and mortality in Pakistan.

## Introduction

Hepatitis B affects 248 million people worldwide, and hepatitis C affects 71.1 million people worldwide [1]. From 1990 to 2017, the number of deaths from hepatitis B increased globally from approximately 580,500 to 799,000, while the global deaths from hepatitis C increased from approximately 350,100 to 580,000 [2]. The hepatitis B virus (HBV) and hepatitis C virus (HCV) are the causative agents in hepatitis B and C, respectively [3,4]. Both HBV and HCV are transmitted by exposure to contaminated blood, and this transfer can be fetal-maternal or via blood transfusion as treatment of different pathologies [5]. Chronic infection with HBV and HCV can lead to cirrhosis of the liver and hepatocellular carcinoma, which leads to morbidity and mortality [6,7].

Pakistan is a low-income country with limited health resources; health care receives only 0.75% of Pakistan’s gross domestic product. In low-income countries like Pakistan, the major risk factor for viral transmission is the use of contaminated medical equipment such as syringes [8]. Heavily populated, low-income nations like Pakistan need strong preventive interventions and extensive management programs to cope with the burden of HBV and HCV patients. Approximately 60% to 80% of HCV-infected patients progress into chronic liver disease [8]. Given that almost 1.5 million people donate blood annually in Pakistan, screening healthy donors will prevent disease and yield data to estimate the disease burden [9,10].

While regional studies have reported prevalence data on HBV and HCV, no studies have assessed the HBC and HCV incidence and trends in Gujranwala. Therefore, this study aimed to screen healthy donors visiting District Headquarters (DHQ) Teaching Hospital Gujranwala to assess disease burden and generate an estimated projection for future prevalence to prepare policymakers for the potential disease burden by 2030.

## Materials and methods

We conducted a retrospective cross-sectional study of blood donor samples collected at DHQ Teaching Hospital in Gujranwala from January 2010 to December 2015. Donors were aged 19 to 60 years, had no chronic diseases (e.g., diabetes, chronic kidney disease, and tuberculosis), and were not in high-risk populations such as sex workers or drug abusers. The donor’s history and eligibility were reported via proforma.

HBV testing

We used a qualitative test based on lateral flow immunoassay using diagnostic kits to test for HBV. We applied the double-antibody “sandwich” technique using a combination of polyclonal and monoclonal antibodies to detect elevated levels of hepatitis B surface antigen for HBV.

HCV testing

We used a diagnostic kit (IHC-302 - HCV Rapid Test Cassette, Vaxpert Inc., Miami, FL) that uses principles of lateral flow immunoassay to detect HCV qualitatively. The membrane of the HCV kit was coated with both antigens and antibodies in the test zone and control zone, respectively. The presence of HCV antibodies produced a dark line in both zones.

Prediction method

Data were analyzed in IBM SPSS Statistics for Windows, version 20.0 (IBM Corp., Armonk, NY) for simple frequencies. Based on data obtained for six years, we predicted the prevalence of HBV and HCV in 2030. To predict 2030 values, we applied the least square method in the time-series analysis of regression. We chose this because a time prediction was needed for the individual variable, and we found it using the following formulas:

ŷ = a + bx

where

b = ∑xy _ (∑x)(∑y)/n

 ∑x2 (∑x)2/n

and

a = ∑y _ b ∑x

 n n

ŷ = dependent variable of the regression line in the least-squares method;

x = independent variable of regression line; and

a and b = estimates of parameters α and β.

The Institutional Bioethics Review Committee approved the study protocols and informed consent documents.

## Results

We screened 66,308 healthy donors in which 715 (1.08%) and 1,846 (2.78%) were positive for HBV and HCV, respectively. We predicted the percentage of HBV and HCV for 2030 to be 3.25% and 6.36%, respectively. The contribution of each year with predicted values for 2030 is presented in Table 1. The predicted value of HBV in 2030 is given in Table 2.

**Table 1:** Prevalence of HBV and HCV in healthy blood donors Abbreviations: HBV, hepatitis B virus; HCV, hepatitis C virus.

Years	Total	Positive HBV, n (%)	Positive HCV, n (%)
2010	10,970	79 (0.072%)	270 (2.96%)
2011	10,813	96 (.0887%)	235 (2.17%)
2012	11,473	118 (1.028%)	299 (2.606%)
2013	10,513	117 (1.11%)	288 (2.74%)
2014	10,691	131 (1.22%)	359 (3.35%)
2015	11,898	174 (1.466%)	395 (3.33%)
Cumulative value	66,308	715 (1.08%)	1,846 (2.78%)
Projection for 2030	12,584	409 (3.25%)	801 (6.36%)

**Table 2:** Calculation for 2030 projected HBV prevalence Abbreviations: HBV, hepatitis B virus.

Years	Y	X	XY
2010	79	−5	−395
2011	96	−3	−288
2012	118	−1	−118
2013	117	1	117
2014	131	3	393
2015	174	5	870
	∑ = 715	0	∑= 579

Values of a and b were found by putting values in the following formulas:

Ŷ = a + bx;

 a = 119, b = 8.3;

 Ŷ = 119.17 + 8.3;

 at 2030, x = 35; and

 Ŷ = 409 (3.25%).

**Figure 1:**
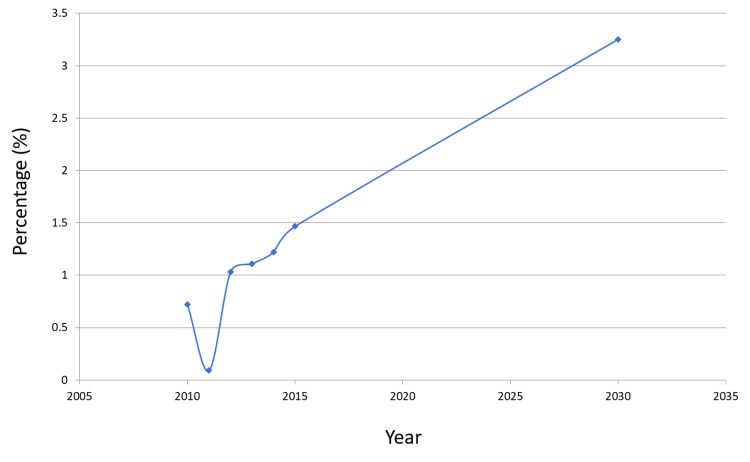
HBV prevalence over time Abbreviation: HBV, hepatitis B virus.

A graphical presentation of the prevalence of HBV over time is manifested in Figure 1. Similarly, the predicted value of HCV in 2030 is presented in Table 3.

**Table 3:** Calculation for 2030 projected HCV prevalence Abbreviations: HCV, hepatitis C virus.

Year	Y	X	XY
2010	270	−5	−1350
2011	235	−3	−705
2012	299	−1	−299
2013	238	1	288
2014	259	3	1,077
2015	395	5	1,975
	∑ = 1,846		∑ = 986

Values of a and b were found by putting values in the following formulas:

Ŷ = a + bx.

As

a = 307.67 and b = 14.09 at 2030, x = 35.

So, 

Ŷ = 801 (6.36%).

**Figure 2:**
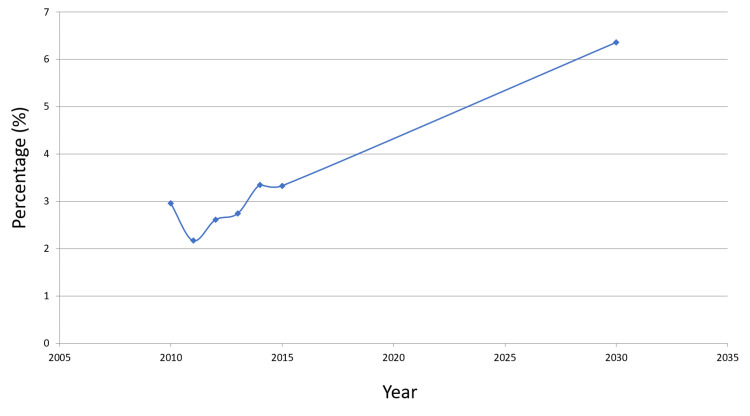
HCV prevalence over time Abbreviation: HBV, hepatitis C virus.

This distribution of HCV over time is shown in Figure 2.

## Discussion

HBV and HCV are viral infections of the liver that disrupt its architecture, causing cirrhosis and hepatocellular carcinoma. HBV and HCV are endemic in Pakistan and inflict a large burden on the national economy. According to the literature, current estimates of the incidence rate of HBV in healthy donors are 2.41% ± 1.96% and 3.31% ± 1.96% for HCV. These data indicate a decreasing trend in HBV prevalence and an increasing prevalence of HCV [11]. In our study, a six-year screening of blood donors found HBV in 1.08% of samples and HCV in 2.78% of samples, with an increasing trend for both viruses except for a slight dip in 2011 in HCV incidence for unknown reasons. The HCV incidence in our study was similar yet slightly higher than other studies in Pakistan. In northern Pakistan, the incidence is 2.3% for HBV and 1.3% for HCV; southern Pakistan has an incidence of 1.7% for HBV and 1.84% for HCV [12,13]. Given that effective treatment exists for HCV, the percentage of HCV infections should be decreasing [14,15]. A lack of awareness and knowledge may be the reason the prevalence is not decreasing.

Our results indicate that the prevalence of HBV is rising, which contrasts with other studies that report a decline in HBV infections due to vaccination rates and the availability of adequate treatment [16,17]. The rising HBV rate we found might be due to inadequate sterilization of surgical instruments, unsterilized syringes, poor awareness of the disease transmission, decreased availability of resources for treatment, and noncompliance of people to treatment and vaccination. Regardless of the reason, the situation is alarming and must be adequately addressed by health care facilities.

By 2030, our results indicate a predicted incidence of 3.25% for HBV and 6.36% for HCV. These are alarming projections and require updated public health policies that work within the limitations of Pakistan’s resources. Through education of both the public and healthcare workers, prevention is a reasonable first goal and could be achieved through seminars, pamphlets, and special training courses. Proper sterilization and efficient screening are other tools to reduce the spread of disease. A well-organized and well-developed vaccination plan is another vital component. The government should provide treatment either free of cost or at a reduced price to make treatment feasible for entire communities.

Our study was limited because our assessment could not depict the actual load of the disease in the community. This is because most of the donors were young men, which excludes older patients, children, and women, all of whom comprise a significant portion of the population. Also, our study did not assess or analyze demographic information. However, our study provides a baseline projection with actionable data that policymakers can use to inform their decisions to help mitigate the rise in HBV and HCV infections that will likely occur if current policies do not change.

## Conclusions

This study projects a growing incidence of HBV and HCV infection in Gujranwala, representing thousands of deaths and reduced quality of life for many people, with a large burden on the economy. Policymakers should use these projections as a baseline and enact changes that will mitigate the expected rise in HBV and HCV infections likely to occur if current policies do not change. Preventive and therapeutic measures are needed to reduce the mortality and morbidity associated with HBV and HBC.
